# Supplementary material for: How Vulnerable Are Patients with COPD to Weather Extremities?—A Pilot Study from Hungary
Source: Healthcare (Basel). 2022 Nov 18;10(11):2309. doi: 10.3390/healthcare10112309 (PMC9690078; doi:10.3390/healthcare10112309)
Supplement: Supplementary file 1 [file healthcare-10-02309-s001.zip › healthcare-2015220-supplementary.pdf]

Supplementary Material: How Vulnerable are Patients with COPD to Weather Extremities? – A Pilot Study from Hungary

Healthcare

Association between extremes of meteorological parameters and the number of ED visits

The risk of increased number of ED visits more than doubled when the maximum temperature was at or below the 10th percentile ($OR_{MXT^{10}}$= 2.690 [CI95% $_{MXT^{10}}$ 1.240 – 5.834]), respectively. The odds ratio of increased number of ED visits was 3.188 (CI95% 1.496 – 6.794), when the minimum temperature was at or below the 10th percentile, and 3.337 (CI95% 1.523 – 7.314), when the average temperature was at or below the 10th percentile. The odds of an increased number of ED visits almost quadrupled ($OR_{STP^{95}}$= 3.985 (CI95% $_{STP^{95}}$ 1.390 – 11.430), when the station level pressure was at or above the 95th percentile; quintupled ($OR_{DP^{10}}$= 4.929 (CI95% $_{DP^{10}}$ 2.149 – 11.305), when the dew point was at or below the 10th percentile. Therefore, the meteorological parameters listed above were risk factors for an increased number of ED visits. (*SM. Figure S1.*)

There was no significant relationship between the 5th, 90th, 95th percentiles of the temperature parameters and the increased number of daily emergency visits. In addition, there was no significant relationship between the 5th, 10th, 90th percentiles of the station level pressure and the increased number of daily emergency visits. No statistically significant relationship was found in the case of precipitation and wind speed either.

The Phi correlation coefficient showed a statistically significant weak relationship between the increased number of ED visits and some of the weather parameters shown in *SI. Table S1*. The coefficients varied between 0.214 (when the dew point was at or below the 10th percentile) and 0.135 (when maximum temperature was at or below the 10th percentile). (*SM. Table S1.*) We did not find a statistically significant relationship between the increased number of ED visits and the rest of the studied weather parameters.

SM. Figure S1. Risk estimation of the effect of extreme meteorological parameters on the number of emergency visits per day.

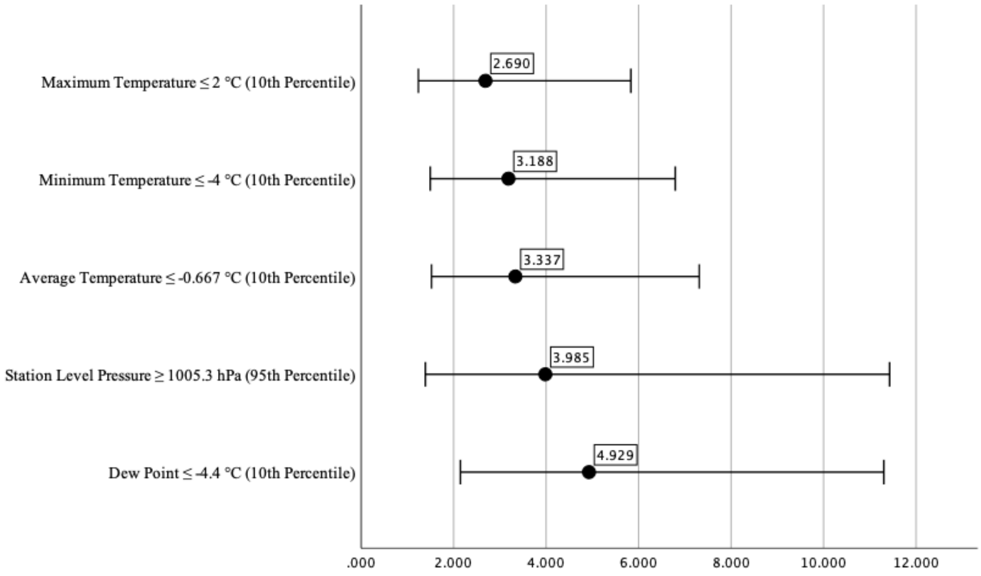

SM. Table S1. Relationship between the extreme meteorological parameters and the number of emergency visits per day, $p < 0.05$.

Weather parameter	Phi correlation coefficient
Dew Point ≤ -4.4 °C (10th Percentile)	0.214
Average Temperature ≤ -0.667 °C (10th Percentile)	0.165
Minimum Temperature ≤ -4 °C (10th Percentile)	0.164
Station Level Pressure ≥ 1005.3 hPa (95th Percentile)	0.144
Maximum Temperature ≤ 2 °C (10th Percentile)	0.135
